# An Uncommon Case of Pediatric Neurobrucellosis Associated with Intracranial Hypertension

**DOI:** 10.1155/2012/492467

**Published:** 2012-07-29

**Authors:** Xenophon Sinopidis, Joseph Kaleyias, Konstantina Mitropoulou, Maria Triga, Sanjeev V. Kothare, Stefanos Mantagos

**Affiliations:** ^1^Department of Pediatric Surgery, Patras University Hospital, University of Patras Medical School, Patras 26504, Greece; ^2^Department of Pediatrics, Patras University Hospital, University of Patras Medical School, Patras 26504, Greece; ^3^Department of Neurology, Boston Children's Hospital, Harvard Medical School, Boston, MA 02115, USA

## Abstract

We present the case of a 4-year-old boy who was admitted to hospital with intracranial hypertension, headache, diplopia, papilledema, and a normal brain MRI. *Brucella melitensis* in the cerebrospinal fluid was confirmed with PCR assay. We believe that neurobrucellosis should be included in the differential diagnosis when headaches persist following brucellosis. In addition, we suggest that when cerebrospinal fluid culture is negative, PCR may prove to be an optimal alternative tool for an immediate and accurate diagnosis.

## 1. Introduction

 Brucellosis is an endemic zoonotic disease, common in certain areas such as the Mediterranean basin, the Middle East, or South America [1, 2]. During the last decades, globalization and technology made worldwide traveling easy. These rather recent population shifts introduced a new era in the epidemic profile of many infectious diseases, changing their distribution, and making them reappear in places where they were previously considered eradicated. For example in 1991, the United Kingdom was declared brucellosis-free yet 148 new cases were reported during the period 2001–2006 amongst them three affecting the central nervous system, otherwise called neurobrucellosis [3]. There are many discrepancies in the diagnostic criteria of neurobrucellosis, and the literature is mainly restricted to case reports and short series [2].

## 2. Case Presentation

 A 4-year-old boy was admitted to our institution because of vomiting and persistent headaches. One month before admission, he had been admitted to another hospital with fever and difficulty walking. Brucellosis is endemic in the region, and Wright agglutination test is routinely performed among other examinations for the differential diagnosis of fever combined with limb dysfunction. The test was positive and the patient was treated by intramuscular gentamicin, oral doxycycline, and rifampicin. Poor compliance to oral antibiotics secondary to vomiting resulted in readmission to our hospital. CBC showed lymphocytic pleocytosis (WBC count 7.400/mL, polymorphonuclear cells 42%, and lymphocytes 48%). Erythrocyte sedimentation rate, liver and renal function tests were normal. The Wright agglutination test for brucellosis in the serum was positive at a titer of 1 : 320. Fundoscopy showed obliteration of the left optic disc margin. An antibiotic regimen of cotrimoxazole and rifampicin was initiated.

 During the first week of admission, headaches worsened. Serial fundoscopy showed frank left side papilledema. Ocular movements revealed left abducens palsy and horizontal diplopia. Papillary light reflexes were bilateral normal and no afferent papillary defect was present. Meningeal or other focal neurological features were absent. Brain magnetic resonance imaging (MRI) was normal, and bone scanning did not reveal any focal involvement.

Lumbar puncture revealed clear cerebrospinal fluid (CSF) with high opening pressure (48 mmHg), and white blood cell count of 162 cells/mL (neutrophils 86%, monocytes 14%), glucose 32 mg/dL, and protein 60 mg/dL. CSF cultures were negative, but CSF polymerase chain reaction (PCR) examination was positive for brucellosis. Hence, the diagnosis of neurobrucellosis associated with intracranial hypertension was confirmed. Ciprofloxacin was added to the initial antibiotic regimen, and acetazolamide was administrated for intracranial hypertension.

 Repeat fundoscopy ten days later showed improvement of the left-sided papilledema. A second measurement of intracranial pressure two weeks later showed an improvement at 33 mmHg, which was further reduced to 26 mmHg after removal of 10 mL of CSF fluid. Headache regressed gradually. One month after admission, fundoscopy showed residual blurring of the temporal margin of the left optic disc. Ciprofloxacin was substituted by moxifloxacine. Acetazolamide was well tolerated and was gradually withdrawn, based on clinical improvement and normal opening pressure measurement on the last lumbar puncture which was performed before discharge.

 After two months in the hospital, the patient was discharged on a regimen of cotrimoxazole, rifampicin, and moxifloxacine for one month, followed by a period of four months with cotrimoxazole and rifampicin, with no residual deficits.

## 3. Discussion

Brucellosis is an endemic infectious disease of animals (zoonosis) that is transmittable to humans through the consumption of their products, after exposure to their blood, or through direct contact [1]. The symptoms of the infection are nonspecific, and often subtle [1]. Diagnosis is suspected if there is a high index of suspicion, due to history of animal product exposure or travelling [1]. A definitive diagnosis is obtained by isolation of *Brucella* species from the blood or other tissues, or by demonstration of high titers of specific antibodies in the serum [1].

Neurobrucellosis ranges from 1.7 to 10% of *Brucella* infections [4, 5]. Incidence in the pediatric age group is much lower, with an average rate of 0.8% [4, 5]. The most common clinical presentation is subacute and chronic meningoencephalitis [3, 7]. Myelitis, radiculoneuritis, brain and epidural abscess, and meningovascular syndromes are other inflammatory processes encountered [2]. MRI examination may present with normal findings, inflammatory changes, or white matter and vascular changes [8, 9].

Leukocyte pleocytosis and high protein levels are seen in the cerebrospinal fluid. Positive cultures from the serum and other specimens are observed in less than 50% [7, 10]. In a recent study of a large number of neurobrucellosis cases, only 28% of blood cultures and 14% of CSF cultures were positive for *Brucella* [2]. As *Brucella* is a slowly growing bacterium, initial cultures in earlier phases of the disease may be negative [3]. The detection of neurobrucellosis is often based on the neurological picture, evidenced by systemic *Brucella *infection, and the presence of inflammatory alteration in the CSF [2]. PCR assay of the cerebral fluid for *Brucella* is a novel evolving and promising diagnostic method [10]. PCR proved to be very helpful in the diagnosis of neurobrucellosis in our case, since CSF cultures were negative.

Involvement of one or more cranial nerves has been noted in more than 50% of neurobrucellosis cases [2, 11]. The main mechanism is basal meningitis affecting the course of the nerves. Other mechanisms include pseudotumor cerebri, vasculitis, and tetracycline side effects [2]. The vestibulocochlear nerve is the most frequently involved [2]. The long intracranial course of the abducens nerve makes it also susceptible to insults like microvascular infarction or direct compression [2]. One case of isolated abducens nerve palsy with borderline intracranial pressure in a young adult female patient has also been reported [11]. Facial, optic, and oculomotor nerves are other reported cranial nerves to be affected [2]. In a pooled analysis of 35 publications on neurobrucellosis in the general population, only one out of 187 (0.5%) patients presented with intracranial hypertension [2].

Papilledema is a common manifestation of intracranial hypertension. In our case this finding was unilateral. Unilateral papilledema is reported in cases of idiopathic intracranial hypertension [12]. The mechanism is unknown. A recent study suggests compartmentation of the subarachnoid space of the optic nerve [13]. Presentation of neurobrucellosis with increased intracranial pressure in children and adolescents is very uncommon (Table 1) [4–6, 14]. In a retrospective study of over 90 children with brucellosis, Tanir et al. reported a 13-year-old female patient with neurobrucellosis, increased intracranial pressure, negative CSF culture, and normal cranial MRI [6]. Yilmaz et al. reported recently a 15-year-old girl with headache, vomiting, diplopia with inward deviation of the left eye, and papilledema. Cranial MRI was normal. Intracranial hypertension was confirmed, and *Brucella melitensis* grew on CSF culture [5].

Our patient presented with intracranial hypertension, headache, diplopia, and papilledema, and normal brain-MRI. *Brucella melitensis* in the CSF was confirmed with PCR-assay. He is the youngest reported case with neurobrucellosis presenting the association of intracranial hypertension, diplopia, and normal cranial MRI. We suggest that neurobrucellosis should be included in the differential diagnosis when headaches persist following Brucellosis. In addition, we also suggest that when CSF culture is negative, PCR may prove to be an optimal alternative tool for an immediate and accurate diagnosis [10].

## Figures and Tables

**Table 1:** Important presenting symptoms, investigation procedures, and treatment features, comparing to other cases of neurobrucellosis presented with intracranial hypertension.

	Sinopidis et al.	Özisik et al. [4]	Yilmaz et al. [5]	Tanir et al. [6]
Patient gender and age	Male 4 years	Female 38 years	Female 15 years	Female 13 years
Clinical presentation	Vomiting Headache Papilledema Horizontal diplopia	Vomiting Diplopia Headache Arthralgia Bilateral papilla stasis	Vomiting Headache Papilledema Horizontal Diplopia	Intracranial hypertension, initially diagnosed as meningoencephalitis, no specific details given
Brain MRI	Normal	Normal	Normal	Normal
Blood examinations	WBC count 7.400/mL Neutrophils 42% Lymphocytes 48% Normal CRP and ESR Wright agglutination test 1: 320	WBC count 5.500/mm^3^ Lymphocytes 64%, Neutrophils 24% Monocytes 12% ESR 20 mm/h Brucella serum test 1/80	Normal blood count Normal CRP ESR 4 mm/h Positive Rose Bengal test Wright agglutination test 1: 160	Serum agglutination test 1: 320 2-Mercaptoethanol test 1: 160
Opening intracranial pressure	48 mmHg	300 mm H_2_O	340 mm H_2_O	Increased, measurement not specified
CSF	162 cells/mL Neutrophils 86% Monocytes 14% Glucose 32 mg/dL Protein 60 mg/dL Negative culture Positive PCR reaction	Lymphocytes 80/mm^3^ Protein 0.20 mg/L Glucose 0.53 mg/L Normal Gram and methylene blue stains Negative ordinary culture Positive BACTEC system culture	10 cells/*μ*L Glucose 58 mg/dL Protein 48 mg/dL Positive Rose Bengal test Wright agglutination test 1: 80 Positive culture	500 cells/mm^3^ Lymphocytes 80% Neutrophils 20% Glucose 47 mg/dL Protein 58 mg/dL Serum agglutination test 1/10 Negative culture
Treatment	Rifampicin, cotrimoxazole, ciprofloxacin added later	Doxycyclin, rifampicin, and trimethoprime with sulfamethoxazole	Streptomycin, doxycyclin, and rifampicin	Doxycyclin, rifampicin, gentamicin
